# Evidence for Porphyrin-Mediated Electron Transfer in the Radical SAM Enzyme HutW

**DOI:** 10.1021/acs.biochem.2c00474

**Published:** 2023-03-06

**Authors:** Marley Brimberry, Patrick Corrigan, Alexey Silakov, William N. Lanzilotta

**Affiliations:** † Department of Biochemistry and Molecular Biology & Center for Metalloenzyme Studies, University of Georgia, Athens, Georgia 30602, United States; ‡ Department of Chemistry, Penn State University, University Park, Pennsylvania 16802, United States

## Abstract

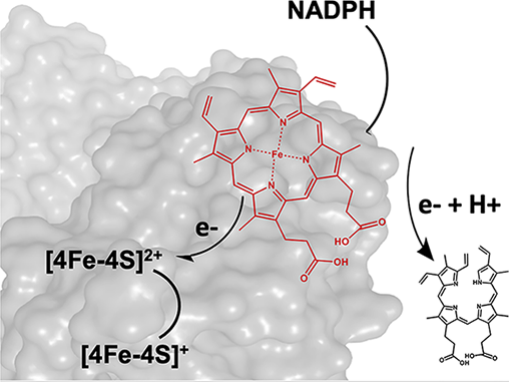

Bacteria that infect the human gut must compete for essential nutrients, including iron, under a variety of different metabolic conditions. Several enteric pathogens, including *Vibrio cholerae* and *Escherichia coli* O157:H7, have evolved mechanisms to obtain iron from heme in an anaerobic environment. Our laboratory has demonstrated that a radical *S-*adenosylmethionine (SAM) methyltransferase is responsible for the opening of the heme porphyrin ring and release of iron under anaerobic conditions. Furthermore, the enzyme in *V. cholerae*, HutW, has recently been shown to accept electrons from NADPH directly when SAM is utilized to initiate the reaction. However, how NADPH, a hydride donor, catalyzes the single electron reduction of a [4Fe-4S] cluster, and/or subsequent electron/proton transfer reactions, was not addressed. In this work, we provide evidence that the substrate, in this case, heme, facilitates electron transfer from NADPH to the [4Fe-4S] cluster. This study uncovers a new electron transfer pathway adopted by radical SAM enzymes and further expands our understanding of these enzymes in bacterial pathogens.

## Introduction

Pathogenic bacteria employ numerous iron-acquisition strategies including the utilization of heme, either free or complexed to heme-containing proteins.^1,2^ Additionally, the regulation of many virulence factors is dependent on iron or heme acquisition from the host.^3−6^ It has been established that once internalized into the bacterium, the iron is liberated from the porphyrin macrocycle through heme oxygenase-type enzymes that catalyze the regiospecific conversion of heme into biliverdin IXα, CO, and free iron.^7^ The discovery of the noncanonical enzymes IsdG and IsdI from *Staphylococcus aureus* as well as MhuD from *Mycobacterium tuberculosis* further expanded the diversity of aerobic heme-degrading systems.^8,9^ Additional research has shown that pathogens such as the enterohemorrhagic *Escherichia coli* O157:H7 and *Vibrio cholerae* liberate iron from heme under anaerobic conditions using a radical *S*-adenosyl-L-methionine (radical SAM or RS) enzyme.^10−12^ It has been postulated that the RS enzyme in *E. coli* O157:H7, ChuW, is responsible for opening the porphyrin ring through a radical mechanism. The product of ChuW is then reduced by an NADPH-dependent reductase, ChuY.^13^ Surprisingly, unlike the bacterium *E. coli* O157:H7, *V. cholera* does not express an enzyme homologous to ChuY. In contrast, we have shown that the RS enzyme HutW can catalyze anaerobic ring opening as well as reduction of the tetrapyrrole directly in the presence of only NADPH.^12^

HutW and ChuW have been identified as class C radical SAM methyltransferases (RSMTs), which employ SAM as a methyl donor in addition to using SAM for radical generation.^14−16^ Members of the RS enzyme superfamily are characterized by their [4Fe-4S] cluster and shared mechanism where reductive cleavage of SAM by the [4Fe-4S] cluster (formally in the +1 oxidation state) results in the formation of methionine and a 5′-deoxyadenosyl radical (5′-dAdo^·^).^17−19^ Mechanistically, this has been proposed to proceed through an organometallic intermediate.^18−22^ RS enzymes are further classified by their domain structure and chemical transformation. In general, class C RSMTs have been shown to methylate an inert sp^2^-hybridized carbon or phosphorus center.^16,23,24^ Both HutW and ChuW have been shown to utilize two molecules of SAM, in a mechanism that is presumably evolutionarily and mechanistically related to HemN.^15^ The first SAM molecule is reductively cleaved by the iron–sulfur cluster to yield the 5′-dAdo^·^, which abstracts a hydrogen atom from the second molecule of SAM to produce a methylene radical.^11,12^ In ChuW and HutW, the methylene radical has been proposed to add to the bridging carbon atom at the *meso* position of the porphyrin ring, resulting in β-scission of the carbon–carbon bond and liberation of the iron atom.^10−12^ While these two enzymes share a mutual mechanistic framework, there are several noteworthy differences, including the electron source and tetrapyrrole product. In *E. coli*, the flavodoxin (EcFldA)/ferredoxin (flavodoxin):NADP+ oxidoreductase (EcFpr)/NADPH system is employed as the electron source.^10,11^ However, neither *E. coli* flavodoxin nor *V. cholerae* flavodoxin was able to reconstitute activity in HutW. Instead, it was discovered that HutW could use NADPH directly as a sole electron source. Furthermore, when NADPH is the reductant, the product of HutW was shown to be a more reduced tetrapyrrole in comparison to the product of the ChuW reaction.^10−12,14,15^

The electron donor seems to be correlated with the catalysis of RS enzymes, and a growing body of research supports RS enzyme activity from diverse electron sources.^25−27^ One example is the RS enzyme MiaB in *Thermatoga maritima*, which does not contain any flavodoxin homologues. Instead, the organism harbors five ferredoxins with a ferredoxin–NADP+ oxidoreductase that maintains the RS enzymatic activity.^25,28^ Another example is TYW1, which was recently shown by Young and Bandarian in *Saccharomyces cerevisiae* TYW1 to encode a flavodoxin-like domain that co-purifies with flavin mononucleotide (FMN) bound, indicating that a redox domain is built into this enzyme.^26^ These observations, when considered in light of NADPH-dependent reduction of HutW, are reminiscent of the family of cytochrome P450s. Genomic sequencing of cytochrome P450s has shown that in addition to two major classes of cytochrome P450 redox partners, there are cytochrome P450 enzymes that are predicted to encode electron delivery subdomains within the same peptide as well as cytochrome P450s that interact directly with hydrogen peroxide or NAD(*P*)H to facilitate oxidative or reductive catalysis.^29^ Cytochrome P450s that have evolved to function without the use of redox partners are the CYP55A P450 subfamily of enzymes, including the prototype enzyme CYP55A1 (P450nor).^30,31^ It seems possible that like cytochrome P450s, there is an evolutionary divergence in the electron transfer mechanisms associated with RS enzymes.

In this work, we further investigate the mechanism of electron transfer in the anaerobic heme-degrading enzyme HutW. Evidence is presented that indicates that the porphyrin ring is essential to trigger oxidation of NADPH and reduction of the [4Fe-4S] cluster. Given that several enteric pathogens encode “W”-type enzymes, with or without the requisite reductase proteins, these data may provide insight into an emerging class of RS enzymes.^17,32,33^

## Results and Discussion

### NADPH Binding

A high affinity for heme has been reported for HutW, but the binding constant for NADPH has not been investigated.^12,14^ To investigate the affinity of NADPH for HutW, we determined the substrate saturation kinetics (*K*_M_^app^) and found HutW to bind NADPH with a *K*_M_^app^ of 7.3 ± 1.1 mM (Supplementary Figure S1). While we acknowledge that this is not a high affinity, it is consistent with what was observed for P450nor and NADPH, which had a *K*_M_^app^ of approximately 4 mM.^34^ Additionally, it indicates that HutW displays saturable kinetics with NADPH. Moreover, given that cytoplasmic NADPH concentrations have been estimated to be anywhere from 0.31 to 22 mM, depending upon cellular conditions,^35,36^ it is reasonable to propose that HutW may utilize NADPH as an electron source when inhabiting an anaerobic environment in the gut.

### Reduction of the [4Fe-4S] Cluster in HutW

A common mechanistic theme for all RS enzymes is that reductive cleavage of SAM requires the [4Fe-4S] cluster to be in the reduced (formally 1+) state. An intriguing observation is that the source of electrons can influence the chemistry of RS enzymes and therefore electron transfer to the catalytic cluster, and subsequent electron/proton transfer steps are of considerable interest toward understanding the mechanism of RS enzymes.^27^

**Figure 1:**
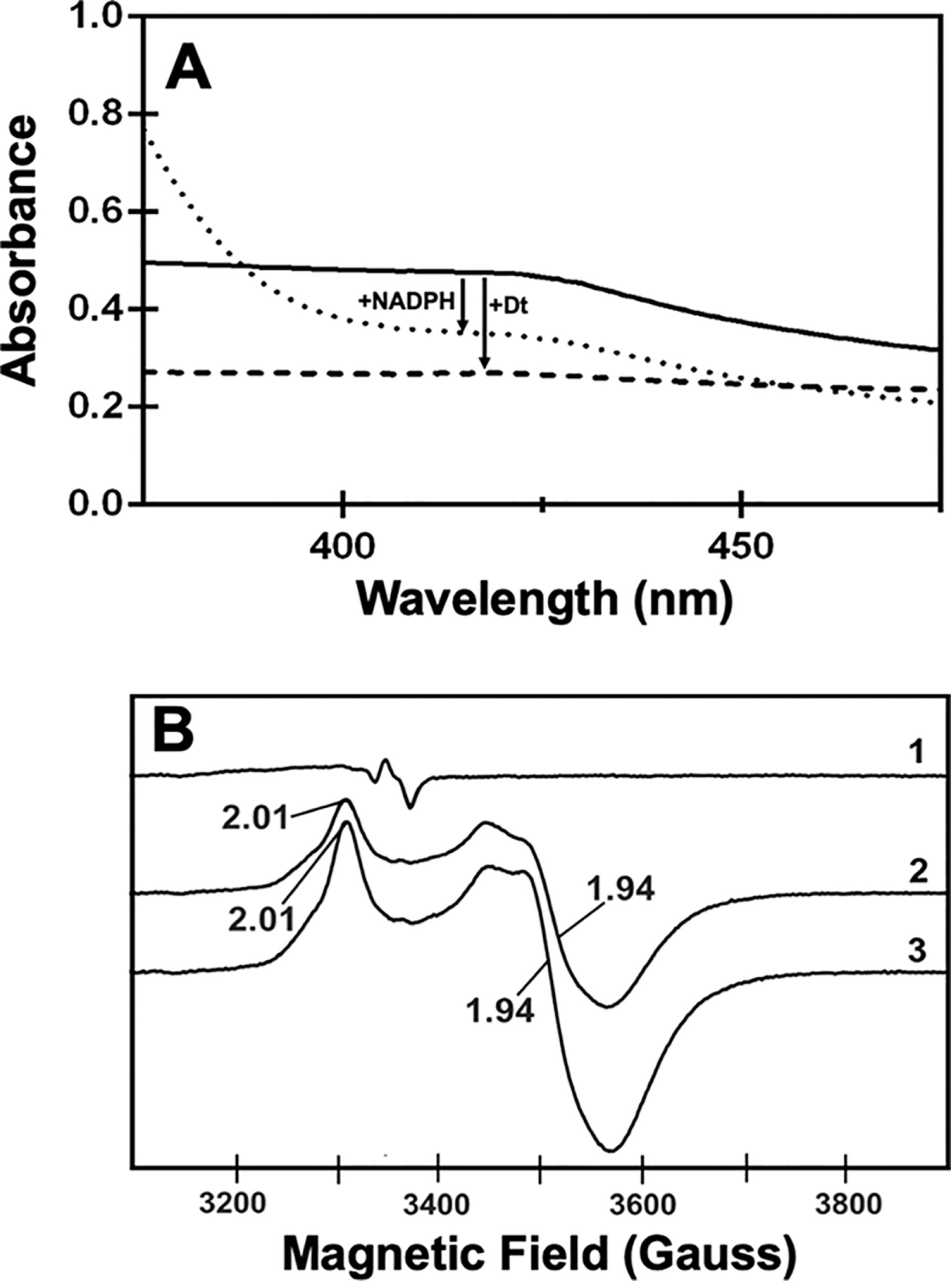
UV–visible (A) and EPR spectroscopy (B) following the reduction of the [4Fe-4S] cluster of as-purified HutW (1) with sodium dithionite (NaDT) (2) or NADPH (3). EPR spectra were recorded at 10 K with a microwave power of 0.1 mW, a modulation amplitude of 1 mT, and a scan time of 180 s, as further detailed in the Materials and Methods section (Supporting Information).

The UV–visible spectrum for as-purified HutW (100 μM), containing an intact [4Fe-4S]^2+^ cluster, reveals a broad absorption band at 400–420 nm (Figure 1A). The addition of a 10-fold molar excess of either sodium dithionite (dashed line) or 5-fold molar excess of NADPH (dotted line) shows bleaching of this band, consistent with the reduction of the [4Fe-4S] cluster. Notably, sodium dithionite (NaDT) appears to result in a greater decrease in the 410 nm adsorption than NADPH (Figure 1A). To provide direct evidence for the reduction of the [4Fe-4S] cluster, we further investigated HutW by electron paramagnetic resonance (EPR) spectroscopy. Consistent with the results from UV–visible spectroscopy, the EPR spectra of NaDT- and NADPH-treated HutW show distinct signals that are typical for a [4Fe-4S]^1+^ cluster, absent in the sample of as-purified HutW (Figure 1B). Therefore, these results confirmed that the presence of NADPH results in reduction of the [4Fe-4S] cluster.

**Figure 2:**
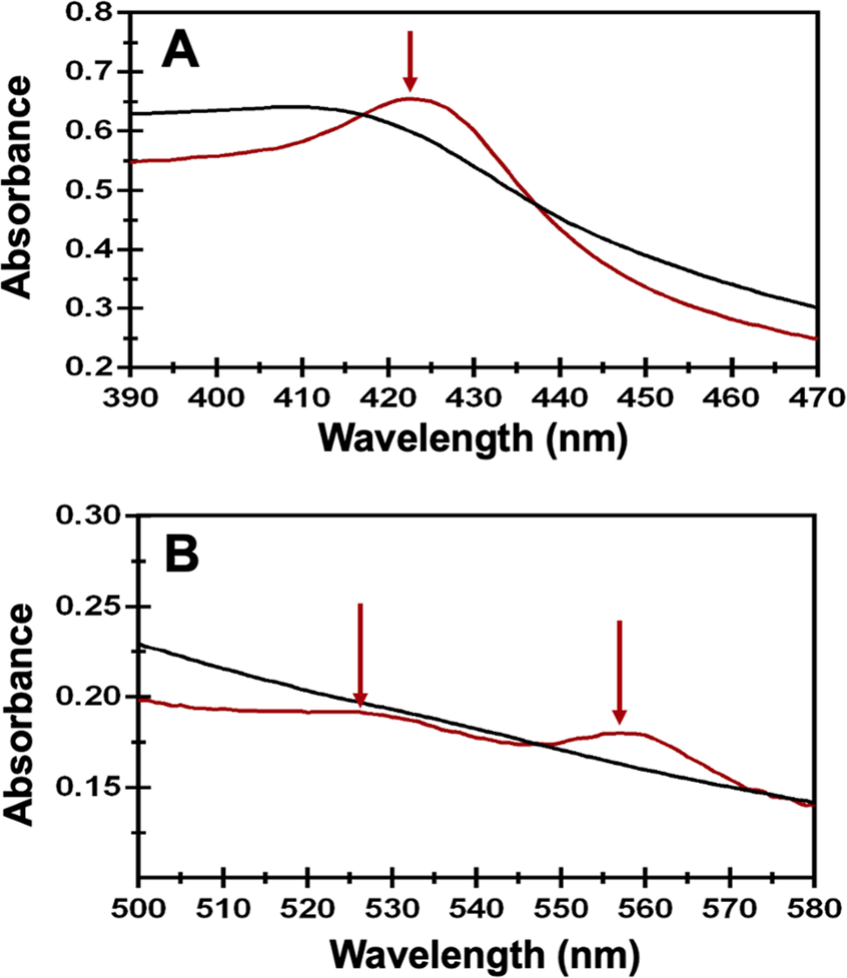
UV–visible spectra highlighting the appearance of a Soret band (A) and β-bands (B) following the addition of sodium dithionite to reconstituted HutW. HutW (100 μM) was isolated with the [4Fe-4S] cluster reconstituted as described in the Materials and Methods section (Supporting Information), and UV–visible spectra were recorded (black line). Sodium dithionite was then added to a final concentration of 1 mM under strictly anaerobic conditions, and another spectrum was recorded (red trace).

Upon closer examination of the UV–visible spectra with and without sodium dithionite, we noticed additional, albeit subtle, spectroscopic changes that are consistent with the reduction of a minor population of heme (Figure 2). Specifically, we observe a shift from a broad absorbance at 420 nm to a distinct peak at 422 nm, indicative of a major γ Soret (Figure 2A). Similarly, longer wavelength features also appeared when excess dithionite was added, as evidenced by the appearance of a β peak at approximately 532 nm and an α peak at 560 nm (Figure 2B). Since the HutW in these experiments is that of a freshly isolated and reconstituted enzyme, these data indicate that some fraction of the prepared protein still contains heme. Likewise, the observed changes in the Soret peak and Q-bands, upon the addition of dithionite, are consistent with the reduction of ferric to ferrous heme and are characteristic of iron in a hexacoordinate complex, likely with two strong axial ligands.^37,38^ Despite what can be extensive isolation strategies, co-purification with heme is a phenomenon that has been observed for other heme- or tetrapyrrole-binding proteins.^39,40^ We were unable to observe an EPR heme signal for heme bound to HutW (Figure 1) discernible by a lack of *g* = 2.83 and *g* = 2.20 for low spin (LS) heme as well as high spin (HS) signal as indicated by a *g* = 6. However, there is some evidence for the latter in concentrated preparations of as-purified HutW (Figure S2). Since there are precedents for hydride transfer from NADPH facilitated through the porphyrin ring,^31,41,42^ we decided to further investigate the effect of the presence of heme on the ability of NADPH to reduce the [4Fe4S] cluster.

### Reduction of the [4Fe-4S] Cluster in Heme-Stripped HutW

To address the possibility that heme was co-purifying with HutW and facilitating electron transfer from NADPH to the [4Fe-4S] cluster, we subjected the enzyme to additional purification steps that would remove any tightly bound heme. One such additional purification step includes treatment with apo-hemoglobin and apo-myoglobin that had been prepared as described previously.^43^ Previous work has shown that bound heme can be removed by incubating the protein of interest with apo-hemoglobin and/or apo-myoglobin because of their high affinity for heme.^44^ Therefore, we treated as-purified (isolated and reconstituted) HutW in this manner to prepare a “heme-stripped” sample of HutW.

**Figure 3:**
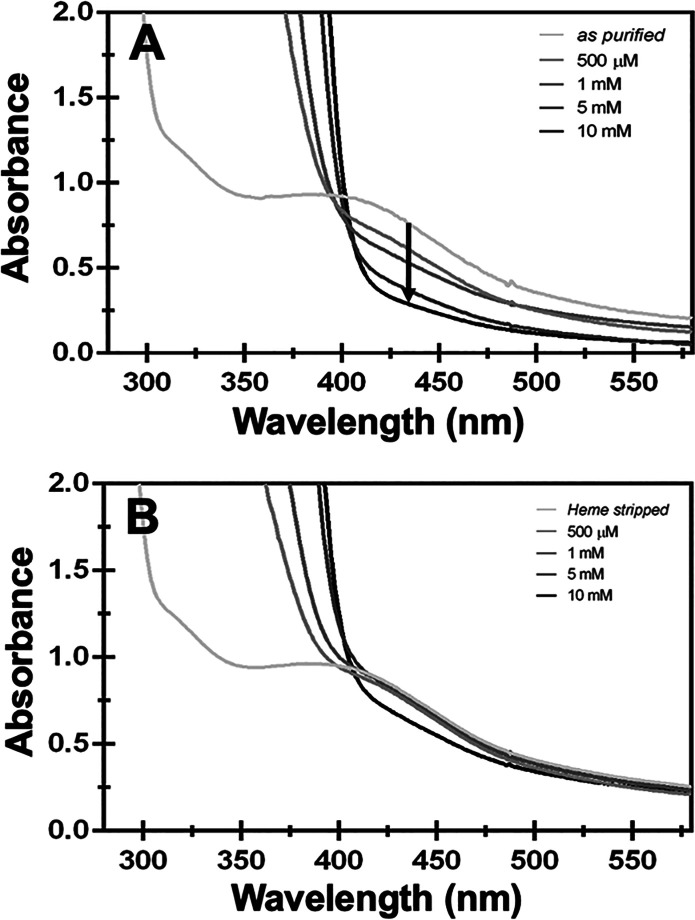
UV–visible spectroscopy of as-purified HutW (A) and heme-stripped HutW (B) titrated with NADPH. As-purified HutW prior to and following treatment with apo-hemoglobin (heme-stripped) was subjected to the addition of NADPH as indicated in the figure panels. Treatment of as-purified HutW with apo-hemoglobin was performed as detailed in the Materials and Methods section (Supporting Information).

To establish if heme may be facilitating reduction of the cluster, increasing amounts of NADPH were injected into a protein sample of either as-purified HutW or heme-stripped HutW, and the UV–visible absorption changes were recorded (Figure 3). The as-purified enzyme shows a reduction in the absorbance of the iron–sulfur cluster within the 400–420 nm range (Figure 3A) when increasing amounts of NADPH are titrated into the sample. However, for heme-stripped HutW, the decrease in absorption upon the addition of NADPH was substantially less (Figure 3B). These data indicate that less reduction has occurred, an observation that is consistent with the proposal that heme binding is required to facilitate the electron transfer from NADPH to the [4Fe-4S] cluster.

**Figure 4:**
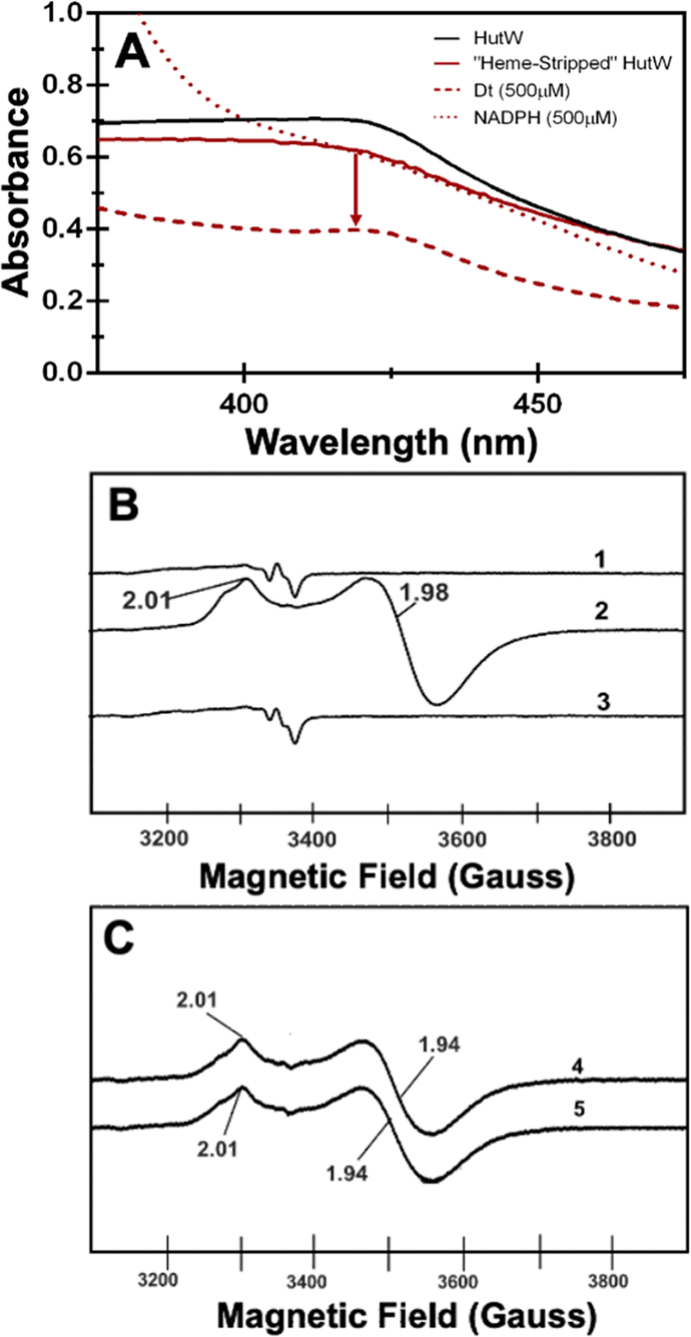
Monitoring the reduction state of heme-stripped HutW by UV–visible and EPR spectroscopy. (A) UV–visible spectroscopy of heme-stripped HutW (solid red trace) following the addition of sodium dithionite (dashed red) and NADPH (dotted red). Reconstituted HutW is shown for comparison (solid black). (B) EPR spectra of heme-stripped HutW without the added reductant (trace 1) and following the addition of sodium dithionite (trace 2) or NADPH (trace 3). (C) EPR spectra of HutW (trace 4) and heme-stripped HutW (trace 5) following the addition of 50 mM heme and NADPH. EPR spectra were recorded at 10 K with a microwave power of 0.1 mW, a modulation amplitude of 1 mT, and a scan time of 180 s, as further detailed in the Materials and Methods section (Supporting Information).

Next, we further validated these results by EPR. Specifically, to address whether the adventitiously bound heme was mediating electron transfer from NADPH to the [4Fe-4S] cluster, we analyzed the heme-stripped HutW sample. The EPR spectra indicate that sodium dithionite is still capable of reducing the [4Fe-4S] cluster in heme-stripped HutW (Figure 4A, trace 2). In contrast, the addition of NADPH to another aliquot of the heme-stripped HutW sample did not result in any reduction of the [4Fe-4S] cluster (Figure 4B, trace 3). In fact, reduction of the cluster by NADPH was only rescued if heme was added back to the sample (Figure 4C). This further supports our hypothesis that the presence of heme is essential for mediating the NADPH-dependent reduction of the [4Fe-4S] cluster in HutW. However, from the evidence presented herein, we are unable to conclusively determine the precise role of heme in mediating the iron–sulfur cluster reduction in HutW. Without evidence of a heme radical intermediate, it is inconclusive whether heme is facilitating the electron transfer itself or simply acting allosterically in the NADPH-dependent reduction of the [4Fe-4S] cluster in HutW. However, it is clear that the EPR spectra of the [4Fe-4S]^+^, whether generated using sodium dithionite and no heme or with NADPH in the presence of heme, show identical spectral features. This suggests that the electronic environment is the same regardless of whether or not heme is present, arguing against an allosteric role for heme. Interestingly, pre-incubation of HutW with 10 mM *S*-adenosyl-l-homocysteine (SAH). appeared to inhibit heme-dependent reduction. This is consistent with observations that SAM or the products of reductive cleavage shift the reduction potential of the cluster to more negative values.^17,18^

## Conclusions

A common mechanistic feature of all RS enzymes is the requirement for the reduction of a [4Fe-4S] catalytic cluster from its resting state of +2 to the active state of +1.^19,45^ The requisite reduction of the [4Fe-4S] cluster prior to catalysis has been shown to be mediated through a variety of redox-active partner proteins.^25,26,28^ Therefore, the discovery that NADPH would function as a direct electron source and catalyze ring opening as well as subsequent reduction of the tetrapyrrole product was surprising but not unprecedented in the context of what is known about heme-dependent enzymes. Specifically, direct electron transfer from NADPH to heme has also been observed for the CYP55A1 cytochrome P450s.^34,41,42^

**Scheme 1:**
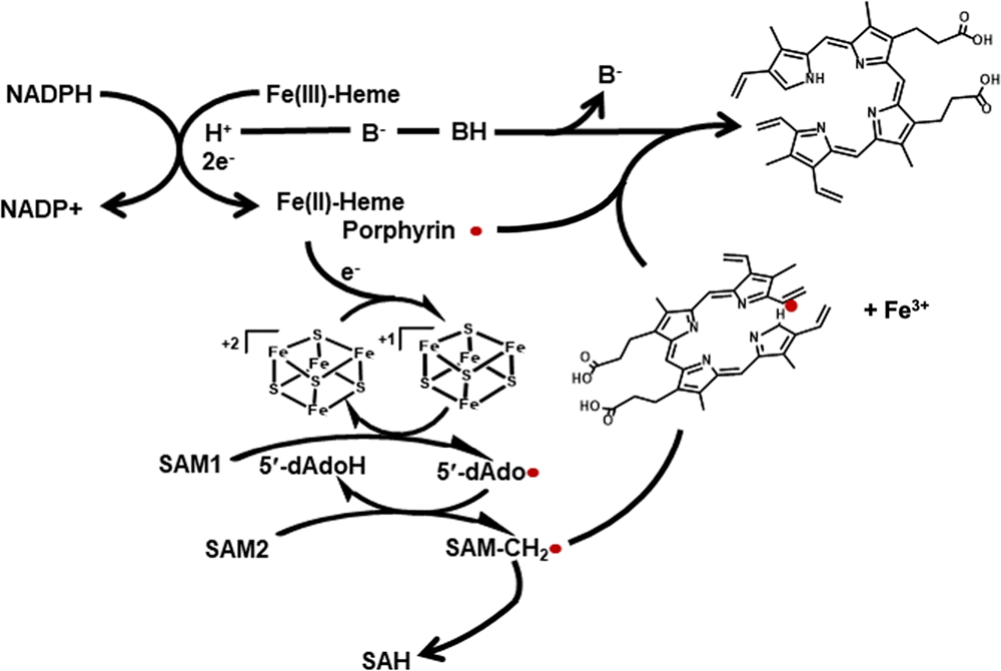
Proposed Mechanism for the Distribution of Reducing Equivalents from NADPH during HutW Turnover

Data presented here allow us to propose a similar heme reduction mechanism in HutW, with the final acceptor of one reducing equivalent being the [4Fe-4S] cluster. We propose that the electron transfer from NADPH to the [4Fe-4S] cluster is mediated by the heme porphyrin ring, similar to mechanisms proposed by others.^46^ Direct electron transfer from NAD(*P*)H to a redox protein that contains only a one-electron redox center is usually impossible because NAD(*P*)H releases two electrons simultaneously as a hydride ion (H−), and a single-electron redox center cannot accept a hydride ion directly.^34^ However, the mechanism for electron transfer from the hydride donor NADPH to reduce the [4Fe-4S] cluster must be rationalized. Therefore, in HutW, we propose that NADPH donates a hydride ion (H−) to the heme substrate, a proposal that is supported by evidence in the cytochrome P450 literature, specifically P450nor and P450BM-3.^47^ In HutW, the hydride is transferred to ferric heme, forming ferrous heme and a porphyrin radical (Scheme 1). The heme porphyrin radical intermediate is currently under investigation but would be transient species that could easily reduce the [4Fe-4S]^2+^ cluster or serve to quench a radical intermediate. The porphyrin propionate or an active site residue could accommodate the proton (shown as “B-” in Scheme 1). Reduction or oxidation of a radical intermediate is another common step in all RS mechanisms, and in this unique case, the properties of the porphyrin substrate and methylated tetrapyrrole intermediate(s) can facilitate electron/proton transfer reactions. Therefore, one rational hypothesis for hydride transfer in HutW, prior to the reduction of the [4Fe-4S] cluster, would be the transient formation of a porphyrin radical (Scheme 1). Overall, these results support the role of the porphyrin ring in mediating electron transfer and alleviating the requirement for a system-specific redox protein. This adaptation potentially facilitates a quicker response to the metabolic needs of the cell and, perhaps, provides an alternative, more direct, regulatory mechanism for this RS enzyme.

## Supporting Information Available

Materials and methods, binding isotherm of for NADPH interaction with HutW, wide-scan EPR spectra for as-purified HutW, and pyridine hemochromagen quantification (PDF)

## Accession Codes

Information on the enzymes being studied in this work can be found under the UniProt IDs A0A3G4VEL8 for HutW.

## Abbreviations

SAM: *S-*adenosylmethionine
RS: radical SAM
EPR: electron paramagnetic resonance
