# Supplementary material for: Evidence for Porphyrin-Mediated Electron Transfer in the Radical SAM Enzyme HutW
Source: Biochemistry. 2023 Mar 6;62(6):1191–6. doi: 10.1021/acs.biochem.2c00474 (PMC10035031; doi:10.1021/acs.biochem.2c00474)
Supplement: Supplementary file 1 — bi2c00474_si_001.pdf [file bi2c00474_si_001.pdf]

Supplementary Information for:

**Evidence for porphyrin mediated electron transfer in the radical SAM enzyme HutW**

*Marley Brimberry[1,3], Patrick Corrigan[2,4], Alexey Silakov[2*] and William N. Lanzilotta[1*]*

[1]Department of Biochemistry and Molecular Biology & Center for Metalloenzyme Studies, University of Georgia, Athens, GA 30602

[2]Department of Chemistry, Penn State University, University Park, PA 16802

[3]Present address: Department of Chemistry, The University of Michigan, Ann Arbor, MI 48109

[4]Present address: Colgate-Palmolive, Piscataway, NJ 08854

*To whom correspondence should be addressed: email wlanzilo@uga.edu or Phone, (706) 202-5622

## MATERIALS AND METHODS

### Overproduction, Purification, and Reconstitution of HutW

As previously described, the gene encoding HutW was codon-optimized for expression in E. coli and synthesized de novo by DNA 2.0 (now ATUM, Newark, CA)[35]. This plasmid utilized a standard T7, isopropyl β-d-1-thiogalactopyranoside (IPTG)-inducible, promoter and was co-transformed along with the pDB1282 plasmid2 containing the *isc* operon, into E. coli BL21(DE3). A 20 mL starter culture of LB medium was inoculated with a single colony and incubated overnight at 37 °C while being shaken at 250 rpm. Flasks (4 L) containing M9 medium (1 L) were inoculated with the overnight starter culture to initiate growth (180 rpm at 37 °C). Expression of the genes encoded on plasmid pDB1282 was induced at an OD600 of 0.3 with 0.2% arabinose. Expression of the *hutW* gene was subsequently induced with 0.2 mM IPTG at an OD600 of 0.6, and the mixture incubated overnight for 18 h at 17 °C while being shaken at 180 rpm. The cells were harvested, flash-frozen, and stored at −80 °C.

For purification, 30 g of frozen cell paste was resuspended in 150 mL of anaerobic lysis buffer containing 50 mM Tris (pH 7.5), 250 mM KCl, 10% (v/v) glycerol, PMSF (1 mM), DNase1 (100 µg/mL), and lysozyme (1 mg/mL). Resuspended cells were degassed with argon while being stirred. Solubilized cells were then lysed anaerobically by equilibrating a closed-system French press with anaerobic buffer while maintaining a stream of argon in the drawing and collection flasks. The lysate was centrifuged at 60000g for 1.5 h. The supernatant was collected and purified anaerobically by a cobalt affinity column pre-equilibrated with buffer. After application of the supernatant, the column was washed with a buffer containing 0 and 10 mM imidazole before

elution with 250 mM imidazole. Protein was diluted to 1 mg/mL, and the [4Fe-4S] cluster was reconstituted following previously established methods. Briefly, the protein was diluted in 100 mM HEPES (pH 8.0), 300 mM KCl, 10% glycerol, and 10 mM DTT. Then, 100 mM ferric chloride was added to 4 times the protein concentration. After the solution had been allowed to incubate for 30 min, 15 μL of 100 mM sodium sulfide was added every 10 min until reaching 1 equiv of the iron concentration. After incubation in the glovebox for 12 h, excess iron–sulfur clusters were removed by centrifugation and the supernatant was run over a DEAE-Sepharose anion exchange column in the glovebox. The reconstituted enzyme was eluted using a stepwise (0.1 M steps) gradient (0-1 M KCl) in buffer. Protein fractions were concentrated anaerobically and applied on a G25 size exclusion column pre-equilibrated with a buffer containing 100 mM HEPES (pH 8.0), 300 mM KCl, and 10% glycerol. Protein fractions were concentrated anaerobically before being stored in liquid nitrogen.

**HutX and HutZ Expression and Purification**

Unlike HutW, HutX and HutZ do not contain any cofactors that would require a unique expression protocol. The gene for HutZ was optimized for expression and cloned into the pD431-SR vector by ATUM. The gene for HutX was amplified from genomic V. cholerae DNA (a generous gift from S. Payne) and cloned into the commercially available pTrcHisA vector. Expression of both proteins was carried out in E. coli BL21(DE3) cells and the aforementioned IPTG-inducible expression plasmids (the HutZ expression vector is kanamycin resistant, while the HutX expression vector is carbenicillin resistant). Expression of HutZ and HutX was performed in LB medium (1 L) inoculated with a 10 mL starter culture and grown at 37 °C while being shaken at 200 rpm. Once an OD600 of 0.6 was achieved, IPTG was added to a final concentration of 1 mM. Cultures were grown for an additional 6 h before being harvested by centrifugation, frozen, and

stored at −80 °C. Frozen cell pellets (50 g) were solubilized in 200 mL of a buffer containing 50 mM Tris (pH 7.5) and 250 mM KCl supplemented with PMSF (1 mM), lysozyme (1 mg/mL), and DNase (1 mg/mL). Cells were lysed aerobically with a French pressure cell, and the lysate was centrifuged at 100000g for 1.15 h. The supernatant was applied to a TALON column that was pre-equilibrated with a buffer containing 50 mM Tris (pH 7.5), 250 mM KCl, and 10% glycerol. The column was washed with the same buffer also containing 0 and 10 mM imidazole buffer before elution with a buffer containing 250 mM imidazole. Protein fractions were analyzed by sodium dodecyl sulfate–polyacrylamide gel electrophoresis (SDS–PAGE) for homogeneity. Protein-containing fractions were pooled and concentrated prior to being frozen at −80 °C.

**General methods and instruments**

All UV-vis spectra were recorded on an HP 8453 diode array spectrophotometer running on OlisWOrks using a Peltier temperature controller set to 35 C and a stir speed of 1200 rpm. Unless indicated in the figure legend, assays were conducted anaerobically with degassed buffer. Turnover was initiated by the addition of SAM unless indicated otherwise. Spectra were recorded from 350 to 900 nm. Graphical figures and data analysis was conducted using GraphPad PRISM.

**Kinetic analysis of HutW reaction**

For the kinetic analysis of HutW, all reaction mixtures contained the following components: 50 mM Tris pH 8.0, 250 mM KCl, 10% glycerol, 5 uM HutW, 10 mM NADPH, 50-250 uM SAM and 10, 15, or 20 uM heme. Specific activity was determined by monitoring the degradation of heme using the extinction coefficient $\varepsilon_{402}$ = 51.63 $mM^{-1}$ $cm^{-1}$ and by the isosbestic point at 418 nm using the relationship A = lε(Csubstrate + Cproduct). Data was fit to the Michaelis-Menten Model followed by a Lineweaver-Burk plot.

**NADPH Binding**

For NADPH binding, all reaction mixtures contained the following components: 50 mM Tris pH 8.0, 250 mM KCl, 10% glycerol, 10 uM HutW, 0.5-22 mM NADPH, 1 mM SAM and 20 uM heme. HutW specific activity was determined by monitoring the degradation of heme using the extinction coefficient $\varepsilon_{402}$ = 51.63 $mM^{-1}$ $cm^{-1}$ and by the isosbestic point at 418 nm using the relationship A = lε(Csubstrate + Cproduct). Data was fit to the one site specific binding model.

**Removal of Heme using apo-Hemoglobin**

Apo-Hemoglobin used in this study was prepared according to the protocol outlined by Ascoli et al.[63] Briefly, 1 mL of a 100 mg/mL Hb solution was rapidly mixed with 20 mL of an acidified acetone solution (24 mM HCl) at 4 °C. The resultant mixture was then centrifuged at 4,800 rpm in a ThermoScientific Sorval S16R tabletop centrifuge for 30 min to pellet precipitated apo-Hb. Pelleted apo-Hb was suspended in 5 M urea and successively dialyzed against 1L ultrapure water for 1 hour followed by overnight dialysis into phosphate buffer. At the end of dialysis, any residual protein precipitate was removed via centrifugation. All samples used for analysis were verified for residual heme by measuring the ratio of absorbance between 404 nm and 280 nm to ensure it was less than 0.1 AU, which indicates less than 1% residual heme 4. Total protein concentration was determined from the 280 nm peak based on the extinction coefficients for apo-Hb found in the literature 4,5.

In an anerobic chamber, HutW was diluted to 10 μM in Tris buffer pH 8.0, 250 mM KCl, 10% glycerol with 5X molar excess apo-Hb and 1 mM sodium dithionite. This solution was allowed to sit overnight after which it was dialyzed multiple times (2 × 1L) in Tris buffer to remove excess sodium dithionite. Proteins were applied to a TALON column. HutW was eluted with 250 mM

imidazole and untagged Hb flowed through. Protein fractions were analyzed by sodium dodecyl sulfate–polyacrylamide gel electrophoresis (SDS–PAGE) for homogeneity. Protein-containing fractions were pooled and concentrated prior to being frozen at −80 °C.

**Table S1. Pyridine hemochromagen quantification for purified, reconstituted HutW.**

| *HutW Purification and reconstitution date* | *Iron Content (Fe/monomer)* | *Heme Content (%)* |
|---|---|---|
| 09/06/21 | 3.6 ± 0.2 | 11.5 ± 0.6 |
| 10/29/21 | 3.4 ± 0.4 | 8.4 ± 0.4 |
| 12/03/21 | 3.2 ± 0.1 | 16.8 ± 0.5 |
| 04/08/22 | 5.9 ± 0.2 | 23.0 ± 0.2 |

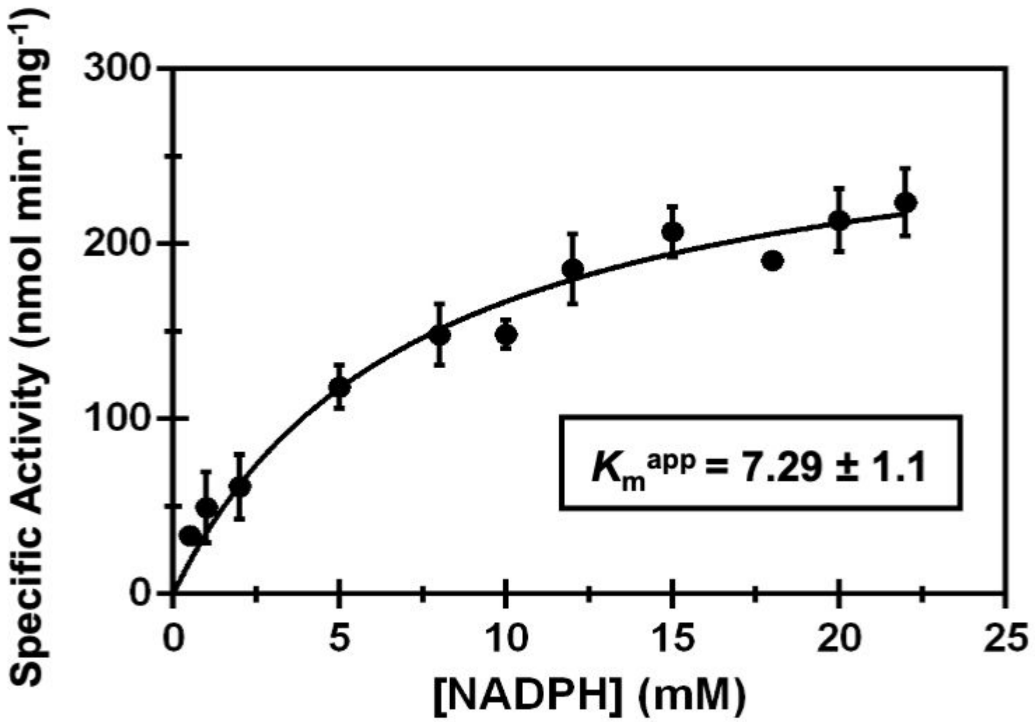


**Figure S1. Binding Isotherm of HutW and NADPH |** HutW specific activity calculated from the disappearance of the heme Soret peak at increasing concentrations of NADPH. Error bars represent the standard deviation of triplicate determinations, with the central point representing the average. $K_M$ was determined using GraphpadPRISM one-state binding kinetics.

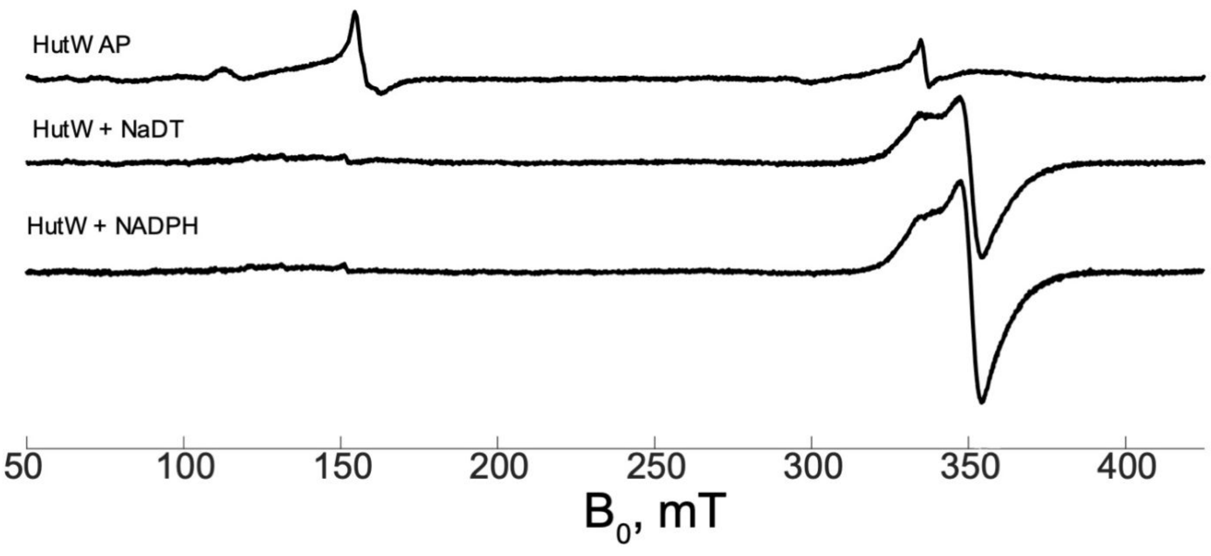


**Figure S2. Wide scan EPR spectra for as-purified HutW (HutW AP), and following the addition of sodium dithionite (HutW + NaDT) or NADPH (HutW + NADPH).** EPR spectra were recorded at 10K with a microwave power of 0.1 mW, and a modulation amplitude of 1 mT. In each case, the spectrum that is shown is the result of four additive scans. Enzyme used in this experiment was purified on 4/8/2022 (Table S1).
